# Th17-skewed immune response and cluster of differentiation 40 ligand expression in canine steroid-responsive meningitis-arteritis, a large animal model for neutrophilic meningitis

**DOI:** 10.1186/s12974-016-0784-3

**Published:** 2017-01-23

**Authors:** Jessica Freundt-Revilla, Arianna Maiolini, Regina Carlson, Martin Beyerbach, Kai Rentmeister, Thomas Flegel, Andrea Fischer, Andrea Tipold

**Affiliations:** 1 Department of Small Animal Medicine and Surgery, University of Veterinary Medicine, Bünteweg 9, 30559 Hannover, Germany; 2 Institute for Biometry, Epidemiology and Information Processing, University of Veterinary Medicine, Hannover, Germany; 3 Tierärztliche Praxis für Neurologie, Dettelbach, Germany; 4 Department of Small Animal Medicine, University of Leipzig, Leipzig, Germany; 5 Clinic of Small Animal Medicine, Centre for Clinical Veterinary Medicine, LMU Munich, Munich, Germany; 6 Center for Systems Neuroscience, Hannover, Germany

**Keywords:** Steroid-responsive meningitis-arteritis (SRMA), Interleukin-17 (IL-17), Cluster of differentiation 40 ligand (CD40L), Interferon gamma (IFN-γ), Cerebrospinal fluid (CSF), Serum, Canine

## Abstract

**Background:**

Steroid-responsive meningitis-arteritis (SRMA) is an immune-mediated disorder characterized by neutrophilic pleocytosis and an arteritis particularly in the cervical leptomeninges. Previous studies of the disease have shown increased levels of IL-6 and TGF-ß_1_ in cerebrospinal fluid (CSF). In the presence of these cytokines, naive CD4+ cells differentiate into Th17 lymphocytes which synthesize interleukin 17 (IL-17). It has been shown that IL-17 plays an active role in autoimmune diseases, it induces and mediates inflammatory responses and has an important role in recruitment of neutrophils. The hypothesis of a Th17-skewed immune response in SRMA should be supported by evaluating IL-17 and CD40L, inducing the vasculitis.

**Methods:**

An enzyme-linked immunosorbent assay (ELISA) was performed to measure IL-17 and CD40L in serum and CSF from a total of 79 dogs. Measurements of patients suffering from SRMA in the acute state (SRMA A) were compared with levels of patients under treatment with steroids (SRMA T), recurrence of the disease (SRMA R), other neurological disorders, and healthy dogs, using the two-part test. Additionally, secretion of IL-17 and interferon gamma (IFN-γ) from the peripheral blood mononuclear cells (PBMCs) was confirmed by an enzyme-linked immunospot (ELISpot) assay.

**Results:**

Significant higher levels of IL-17 were found in CSF of dogs with SRMA A compared with SRMA T, other neurological disorders and healthy dogs (*p* < 0.0001). In addition, levels of CD40L in CSF in dogs with SRMA A and SRMA R were significantly higher than in those with SRMA T (*p* = 0.0004) and healthy controls (*p* = 0.014). Furthermore, CSF concentrations of IL-17 and CD40L showed a strong positive correlation among each other (rSpear = 0.6601; *p* < 0.0001) and with the degree of pleocytosis (rSpear = 0.8842; *p* < 0.0001 and rSpear = 0.6649; *p* < 0.0001, respectively). IL-17 synthesis from PBMCs in SRMA patients was confirmed; however, IL-17 is mainly intrathecally produced.

**Conclusions:**

These results imply that Th17 cells are inducing the autoimmune response in SRMA and are involved in the severe neutrophilic pleocytosis and disruption of the blood-brain barrier (BBB). CD-40L intrathecal synthesis might be involved in the striking vasculitis. The investigation of the role of IL-17 in SRMA might elucidate important pathomechanism and open new therapeutic strategies.

## Background

Steroid-responsive meningitis-arteritis (SRMA) is the most frequently diagnosed meningitis in canines [1]. It is a systemic immune-mediated disorder [2] characterized by systemic inflammatory lesions of the vessels, but particularly in the cervical leptomeninges [3, 4] and in a recognized large animal model for neutrophilic meningitis [5]. This disorder affects typically young adult dogs [6], can occur in any breed, although beagles, boxers, Bernese mountain dogs [3, 7], Weimaraners, Nova Scotia duck tolling retrievers [2], and Petit Basset Griffon Vendéen [8] are over-represented.

Two different forms of SRMA are recognized, a typical acute form and a protracted atypical form [7]. In the acute one, common clinical signs include fever, reluctance to move, stiff gait, cervical rigidity, and pain [3]; the analysis of cerebrospinal fluid (CSF) reveals a marked polymorphonuclear pleocytosis and elevated protein [7]. The protracted form may be observed following relapses and neurological deficits like reduced menace response, anisocoria, strabismus, and variable degrees of paresis and ataxia might occur; CSF analysis at this stage reveals mononuclear or mixed cell populations [7].

The etiology of SRMA remains unknown, even a genetic predisposition was described in Nova Scotia duck tolling retrievers [9]. Up to now, no infectious agents eliciting the disease have been consistently detected [4, 6, 7, 10–12]. Several studies [13–17] confirmed an immune-mediated disease. Furthermore, high concentrations of immunoglobulin A (IgA) have been found both intrathecally and systemically in dogs affected with SRMA [13, 18].

A predominance of T helper lymphocytes (CD4+) in the peripheral blood [15] with a prominent Th2-mediated immune response in dogs suffering from SRMA was shown [17]. Furthermore, increased levels of interleukin 6 (IL-6) and transforming growth factor *beta* 1 (TGF-ß_1_) have been found in CSF. A combined intrathecal increase of these proteins could induce CD4+ progenitors to differentiate to the recently discovered third T helper subset (Th17) and enhance the autoimmune response in SRMA [14].

Interleukin 17 (IL-17) synthesizing cells are known to be involved in the pathogenesis of several autoimmune diseases [19–22]. IL-17 is a pro-inflammatory cytokine secreted primarily not only by activated T cells [23] but also by neutrophils [24], eosinophils [25], and monocytes [26] and acts on the IL-17 receptor (IL-17R) [27]. Furthermore, IL-17 has a role, coordinating local tissue inflammation through the induced release of pro-inflammatory and neutrophil-mobilizing cytokines [28, 29]. It has been shown to disrupt tight junctions between the endothelial cells of the blood-brain barrier in humans [30]. These mechanisms might explain an influence of IL 17 on the massive invasion of neutrophils into the subarachnoidal space in dogs affected with SRMA.

The CD40 Ligand or CD154 is primarily expressed on the surface of activated CD4+ T cells [31]. However, activated T cells not only express CD40L on their membranes, but a soluble, biologically active form of CD40L also interacts with the CD40 receptor [32] expressed mainly by B cells but also by many other cells [31]. The vascular endothelial cells express the CD40 receptor, and its interaction with CD40L leads to endothelial cell activation and leukocyte adhesion [33].

In the current study, we hypothesized the following: (1) that a Th17-skewed immune response is involved in the inflammatory reaction in SRMA and should be confirmed by evaluating IL-17 systemically, intrathecally, and at the cellular level; (2) that a dysregulated expression of CD40L is presumably involved in the pathogenesis of SRMA. Upon confirmation of a Th17-skewed immune response, new treatment strategies directly influencing this cell population could be evaluated in this model.

## Methods

### Serum and cerebrospinal fluid samples

Ninety-eight serum and 98 CSF samples were collected between 2005 and 2015, aliquoted and stored at −20 °C until determination of IL-17 and CD40L concentrations by a sandwich enzyme-linked immunosorbent assay (ELISA) was performed. Samples were taken from client-owned patients and controls derived from healthy university-owned beagles of the University of Veterinary Medicine Hannover, Germany. This study was conducted in accordance with the ethical guidelines of the University of Veterinary Medicine Hannover and was approved by the authorities of Lower Saxony (Animal experiment number 33.9-42502-05-14A453). CSF samples were collected by suboccipital puncture of the dogs under general anaesthesia, and serum samples were obtained by puncture of the cephalic or saphenous peripheral vein. All control dogs had routine CSF parameters in physiological ranges relating to the cell count (0–3 cells/μl), glucose (60–80% of the blood glucose concentration), and protein (less than 25 mg/dl) [34]. CSF samples with severe blood contamination were excluded, and in ten inflammatory CSF samples, a slightly elevated erythrocyte count was detected reflecting the vasculitis in SRMA [35]. Clinical and neurological examinations in all the healthy control dogs revealed no pathological findings. Client-owned patients were divided into four groups: SRMA in the acute stage (SRMA A), SRMA showing relapses of the disease (SRMA R), SRMA under treatment with glucocorticosteroids (SRMA T), and miscellaneous (neoplasia, intervertebral disc herniation (IVDH), fever of unknown origin, and meningoencephalitis of unknown origin (MUO).

SRMA in the acute stage was defined by occurrence of cervical rigidity and pain, fever, and polymorphonuclear pleocytosis in the CSF. IgA content in serum and CSF was determined to support diagnosis of SRMA as previously described [7]. Other possible causes of neck pain, elevated body temperature and/or pleocytosis were ruled out. Patients in the SRMA A group were not pre-treated with glucocorticosteroids prior to CSF puncture. Clinical signs, previous/ongoing treatment and CSF parameters for the three stages of the disease (SRMA acute, SRMA relapse and SRMA under treatment) at the time of sampling were considered as previously described [14].

### EDTA blood samples

Fresh ethylenediaminetetraacetic acid (EDTA) blood samples were prospectively collected from 18 healthy university-owned beagles and 96 client-owned patients suffering from different neurological disorders between 2014 and 2015 at the Department of Small Animal Medicine and Surgery, University of Veterinary Medicine Hannover, Germany. The diagnosis of the neurological disorder was performed by a resident or a diplomate of the European College of Veterinary Neurology (ECVN). Diagnoses were based on clinical signs, neurological examination, complete blood cell count, blood chemistry, CSF analysis, different imaging techniques, electrophysiology, surgery, and/or histopathology when needed. EDTA blood was immediately processed after sampling for isolation of the peripheral blood mononuclear cells (PBMCs), which were subsequently used for the ELISpot assays.

### PBMCs isolation and freezing

The mononuclear cell fraction was separated by density gradient centrifugation, using Histopaque®-1.199 (N° 11191, Sigma-Aldrich®, Saint Louis, USA) and Pancoll human 1.077 (N° P04-60500, Pan Biotec™) as described before [36]. Isolated PBMCs were washed three times with phosphate-buffered saline (PBS buffer: 8.00 g NaCl, 0.20 g KCl, 1.15 g Na_2_HPO_4_, 0.20 g KH_2_PO_4_, in 1000-ml ultrapure water, pH 7.4). When the cell pellet was contaminated with erythrocytes, hypotonic lysis was performed using purified water and double-concentrated PBS. Subsequently, the number of viable cells was determined by staining with Tryptan blue solution 0.4% (N° T6146, Sigma-Aldrich®, Saint Louis, USA) in PBS. The cells were resuspended and diluted to a concentration of 40 × 10^6^ cells/ml in an ice-cold Roswell Park Memorial Institute 1640 medium with l-glutamine phenol red (RPMI 1640 Medium, 21875-034, Gibco®) and 15% serum replacement-1 (S0638, Sigma-Aldrich®, Saint Louis, USA). The following steps were performed on ice: 0.5 ml of cell suspension was dispensed in labelled cryovials and ice-cold RPMI 1640 medium with l-glutamine, 15% serum replacement-1, and 20% dimethyl sulfoxide (DMSO, Sigma-Aldrich®, Saint Louis, USA) was added slowly to obtain a final cell suspension of 20 × 10^6^ cells/ml. Finally, the cryovials were placed immediately in a freezing container (N°5100-0001, Fisher Scientific GmbH, Schwerte, Germany) and stored at −90 °C. After >24 h the cryovials containing the cell suspension were stored at −150 °C until further analysis.

### Determination of IL-17 and CD40L concentrations in CSF and serum

Both proteins were measured in CSF and serum samples using sandwich enzyme-linked immunosorbent assay commercial kits to detect canine IL-17 (SEA063Ca, Cloud-Clone Corp., Houston, USA) and canine CD40L (SEA119Ca, Cloud-Clone Corp., Houston, USA), following the manufacturer’s protocols. Briefly, CSF, serum samples, and standards of the tested proteins were diluted to the desired dilutions. Samples were diluted in 0.01 mol/L PBS, pH 7.0–7.2. Final dilution for most SRMA A and SRMA R samples was of 1:8 for IL-17 in CSF as most of these samples exceeded the maximal detectable value. Standard or samples were added in duplicates (100 μl per well) in 96-well pre-coated plates and incubated for 2 h at 37 °C. Subsequently, the liquid of each well was removed followed by incubation with 100 μl per well of detection agent A (biotin-conjugated antibody specific to canine IL-17 or canine CD-40L) for 1 hour at 37 °C. After washing three times, the remaining wash buffer was removed by decanting and 100 μl per well of detection agent B (avidin conjugated to horseradish peroxidase) were added to each well and incubated for 30 min at 37 °C. After adequate washing steps, 90-μl/well TMB chromogenic substrate (3.3′.5.5′ Tetramethylbenzidine) was added and incubated for 25 min at 37 °C. After adding 50-μl stop solution containing sulphuric acid, the plates were measured using a microplate reader (Synergy 2 Multi-mode microplate reader, BioTek Instruments Inc., Bad Friedrichshall, Germany) spectrophotometrically at a wavelength of 450 nm ± 10 nm.

All samples were tested in duplicates and the mean value was calculated. The minimum detectable value for IL-17 was 7.8 pg/ml and for CD40L 0.156 ng/ml, lower values were considered negative (0 pg/ml and 0 ng/ml, respectively). Samples resulting in measurements exceeding the maximal detectable value for IL-17 (500 pg/ml) or CD40L (10 ng/ml) were diluted and measured again.

### Determination of IL-17 and IFN-γ-producing PBMCs

Quantitative determination of the frequency of PBMCs releasing canine IL-17 and/or interferon gamma (IFN-γ) was performed using a commercially available dual-Color enzyme-linked immunoSpot (ELISpot) assay (N° ELD6555, R&D Systems®, Inc., Minneapolis, USA), following the manufacturer’s instructions. Briefly, all reagents were brought to room temperature except for the detection antibody concentrate and the dilution buffer. Cells to be analysed were thawed and washed with RPMI 1640 medium once the number of viable cells was determined and two different dilutions were made for each sample (2.5 × 10^5^ cells/ml and 5 × 10^5^ cells/ml) in RPMI 1640 medium containing l-glutamine, 15% serum replacement-1, and 4-μg/ml concanavalin A (N° C5275, Sigma-Aldrich®, St. Louis, USA) for stimulation. Simultaneously, all wells from a 96-well PVDF (polyvinylidine fluoride) membrane-backed microplate coated with a monoclonal antibody specific for canine IFN-γ and a monoclonal antibody specific for canine IL-17 were filled with 200-μl RPMI 1640 medium containing l-glutamine and incubated for 20 min at room temperature. When the cells were ready to be plated, the medium of each well was removed and 100 μl of appropriate controls and PBMCs in stimulation medium were added to each well. All samples and controls were assayed in triplicate. The cells were stimulated overnight at 37 °C in a 5% CO_2_ incubator. After incubation, the wells were washed four times with wash buffer (300 μl pro well) to remove the cells and culture medium and 100-μl detection antibody mixture (containing biotinylated polyclonal antibody specific for canine IFN-γ and horseradish peroxidase-conjugated polyclonal antibody specific for canine IL-17) was added to each well and incubated at 4 °C overnight. After incubation, the liquid of each well was removed by aspiration and washing was performed four times with wash buffer. One hundred microliter streptavidin-AP (streptavidin conjugated to alkaline phosphatase) was added to each well and incubated for 2 h at room temperature. After adequate washing steps, 100 μl of BCIP/NBT chromogen [5-bromo-4-chloro-3′indolylphosphate p-toluidine salt (BCIP) and nitro blue tetrazolium chloride (NBT)] were added to each well and incubated for 1 h protected from light at room temperature. BCIP/NBT chromogen was discarded and plates were rinsed once with deionized water. After removal of water was completed, 100 μl AEC (3-amino-9-ethylcarbazole) chromogen in stabilizing buffer (0.1% H_2_O_2_ in acetate buffer) were added to each well incubating for 20 min protected from light at room temperature. After decanting and rinsing with deionized water, excess of water and plate underdrain were removed. Finally, microplates were dried completely at room temperature for 90 min before analysis.

**Fig. 1:**
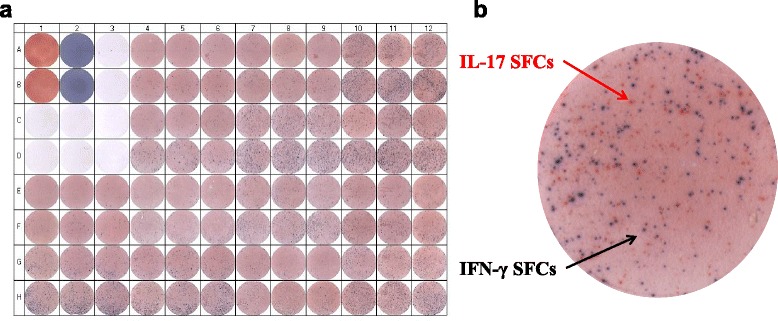
IL-17 and IFN-γ spot-forming cells. **a** Scan of a 96-well PVDF-backed ELISpot microplate. Positive and negative controls are shown *vertically* in duplicates (A1-D3) and sample wells *horizontally* in triplicates (E1-H12). **b** *Red spots* (IL-17-producing PBMCs) and *black spots* (IFN-γ-producing PBMCs) are presented in a *zoomed well* (C10). Brightness: 75% and region of interest (ROI): 66%

An ELISpot puncher (Eli.Punch, A.EL.VIS GmbH, Hannover, Germany) was used to remove the 96-well bottoms, and counting of spots was performed using an ELISpot scanner and Analysis Software (Eli.Scan, A.EL.VIS GmbH, Hannover, Germany). All samples were tested in triplicates for both dilutions (2.5 × 10^5^ cells/ml and 5 × 10^5^ cells/ml). Since the wells from the 5 × 10^5^ cells/ml dilution showed a high density of the spots and the 2.5 × 10^5^ cells/ml dilution showed better defined, distinct, separated, clear spots, only this dilution was further analysed. Red spots (IL-17-producing PBMCs) and black spots (IFN-γ-producing PBMCs) were counted separately, and the settings used were brightness: 75% and region of interest (ROI): 66% (Fig. 1). Results are expressed in number of spot-forming cells (SFC) per number of cells in each well. Finally, the means from the well triplicates were calculated.

### Statistical analysis

Statistical evaluation to test for significant differences of IL-17 and CD40L concentrations in CSF and serum, and IL-17- and IFN-γ-producing PBMCs, among the different groups, was performed using SAS® software Version 9.3 (SAS Inst. Inc. Cary, NC, USA). Graphics from the statistical data obtained were made by using GraphPad software (GraphPad Prism®, version 5, La Jolla, CA, USA).

### Statistical analysis of IL-17 and CD40L concentrations in CSF and serum

In the statistical analyses, IL-17 and CD40L values under the detection limits were treated like zeros and will be referred to as “zero values” hereinafter. All groups were compared pairwise using the two-part test [37, 38]. This test combines the comparison of the percentages of zeros using Fisher’s exact test and Wilcoxon’s two-sample test of the values above the detection limits (non-zero values). When not only zero values were present in one or both groups but also values greater than zero in both groups, then the two-part test was used. However, if there were only zeros in a group, only the Fisher exact test was used. Furthermore, the two-part test was not necessary for the analysis of CD40L in serum, since no zeros were present in this variable. Therefore, the Wilcoxon test alone was performed for this analysis given that the non-zero values were not normally distributed. Results are given as medians, minimal and maximal values of the non-zero values, and percentages of zero values. Additionally, Spearman’s rank correlation coefficients (rSpear) were calculated to determine correlations between IL-17 and CD40L and between white blood cell count in CSF and both proteins. A comparisonwise error rate of 5% was applied in the analysis of the data, differences between groups and correlation coefficients were claimed as significant if the corresponding *p* values were lesser than 0.05.

Given the prolonged period of storage of some serum and CSF samples, linear regression analyses separated by groups were performed to look for significant linear regression coefficients of IL-17 and CD40L through the years where the samples were collected. No significant correlations between the concentration of the proteins analysed and the years of sampling were found in any of the groups (*p* > 0.05).

### Statistical analysis of IL-17 and IFN-γ-producing PBMCs

Since these variables were not normally distributed within the groups, all groups were compared pairwise using the Wilcoxon two-sample test. Medians of the means were compared and values of *p* < 0.05 were considered significant.

## Results

### Serum and CSF samples

Serum and/or CSF were collected and analysed from 79 dogs in total. In some dogs with SRMA, samples were taken twice, in the acute phase (SRMA A) and during treatment (SRMA T).

From the total of 98 CSF and serum samples, 89 were paired CSF and serum samples. In some cases, both proteins could not be measured because the material was not sufficient (Table 1).

**Table 1:** Number of CSF and serum samples analysed for IL-17 and CD40L in each group

Groups	IL-17		CD40L	
	CSF	Serum	CSF	Serum
SRMA A	30	30	31	31
SRMA R	6	6	6	6
SRMA T	26	27	26	28
Healthy	12	14	14	14
Miscellaneous	8	9	8	8
Total	82	86	85	87

*IL-17*, interleukin-17, *CD40L* cluster of differentiation 40 ligand, *CSF* cerebrospinal fluid, *SRM A* steroid-responsive meningitis-arteritis in acute stage, *SRMA R* recurrence of SRMA, *SRMA T* SRMA patients under treatment

Among the patients with SRMA, 16 different breeds and mixed-bred dogs were included. The most common breeds were boxer (25%), Bernese mountain dogs (16.7%), beagles (16.7%), and mixed breeds (10.4%). Most dogs diagnosed with SRMA in the acute phase of the disease were <1 year (55.2%), 34.48% were between 1- and 2-years old, and 10.3% were between 2- and 3-years old.

### CSF and serum concentrations of IL-17

The highest concentrations of IL-17 were found in CSF samples of patients with SRMA A (median 901.77 pg/mL; range 70.73–2967.14 pg/mL; percentage of zeros 0%), followed by those with SRMA R (median 533.01 pg/mL; range 97.52–1771.87; percentage of zeros 0%). Concentrations of IL-17 CSF samples “SRMA A” differed significantly from other groups, SRMA T (*p* < 0.0001), healthy dogs (*p* < 0.0001), and miscellaneous (*p* = 0.0005). However, there was no difference to values measured in samples from dogs with relapses (SRMA R), a group also displaying significant differences with SRMA T (*p* = 0.0004), healthy dogs (*p* = 0.0051), and miscellaneous (*p* = 0.0415).

Similarly, the highest values of IL-17 in serum were found in dogs with SRMA A (median 45.95 pg/mL; range 12.18–251.53 pg/mL; percentage of zeros 50%), followed by serum samples of dogs with SRMA R (median 43.30 pg/mL; range 12.52–126.74 pg/mL; percentage of zeros 16.67%). Significantly higher values were found in SRMA A when compared with the group “miscellaneous” (*p* = 0.0445). Furthermore, statistically significant differences were found between the groups “SRMA R” and “healthy” (median 32.34 pg/mL; range 9.40–87.46 pg/mL; percentage of zeros 14.29%) when compared with SRMA T (*p* = 0.0166 and *p* = 0.0021, respectively) and miscellaneous (*p* = 0.0268 and *p* = 0.0022, respectively).

**Fig. 2:**
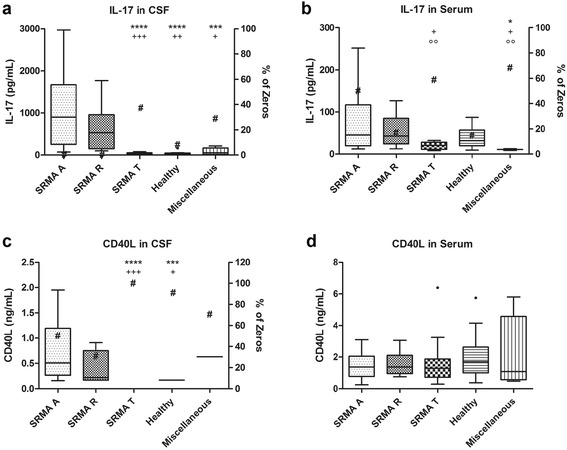
Concentration of IL-17 (**a**, **b**) and CD40L (**c**, **d**) in CSF and serum samples and percentage of zeros. *Boxes* contain values from the first to the third quartile, *lines inside the box* indicate median values, *endpoints of vertical lines* represent minimum and maximum values, and • represent outliners. *Number sign* represents percentage of zero values. *Asterisks* indicate statistically significant differences from the SRMA A group (****p < 0.0001, ***p < 0.005), *plus* indicate statistically significant differences from the SRMA R group (^+++^ *p* < 0.005, ^++^ *p* < 0.01, ^+^ *p* < 0.05) and *circles* indicate statistically significant differences from the healthy group (^oo^ *p* < 0.01). IL-17: interleukin-17, CD40L: cluster of differentiation 40 ligand, CSF: cerebrospinal fluid, SRMA: steroid-responsive meningitis-arteritis, SRMA A: acute stage of SRMA, SRMA R: recurrence of SRMA, SRMA T: SRMA patients under treatment

IL-17 concentrations in CSF and serum, in addition to percentage of zeros (values under the detection limits), are summarized in Table 2. Significant differences among groups are shown in Fig. 2.

**Table 2:** Concentrations of IL-17 and CD40L in CSF and serum samples analysed

Groups		IL-17 (pg/mL)		CD40L (ng/mL)	
		CSF	Serum	CSF	Serum
SRMA A	Median	901.77	45.95	0.51	1.37
	Range	(70.73–2967.14)	(12.18–251.53)	(0.16–1.95)	(0.25–3.10)
	Percentage of zeros	0%	50%	50%	–
SRMA R	Median	533.01	43.30	0.22	1.37
	Range	(97.52–1771.87)	(12.52–126.74)	(0.17–0.91)	(0.76–3.06)
	Percentage of zeros	0%	16.67%	33.33%	–
SRMA T	Median	29.67	21.04	0	1.29
	Range	(12.47–78.86)	(8.10–32.92)	(0–0)	(0.29–6.39)
	Percentage of zeros	38.46%	59.26%	100%	–
Healthy	Median	30.90	32.34	0.17	1.70
	Range	(15.45–172.17)	(9.40–87.46)	(0.17–0.17)	(0.38–5.75)
	Percentage of zeros	8.33%	14.29%	92.86%	–
Miscellaneous	Median	53.54	10.98	0.63	1.08
	Range	(13.64–221.82)	(8.65–13.30)	(0.63–0.63)	(0.48–5.80)
	Percentage of zeros	30%	69.23%	72.73%	–

Median and range (minimum-maximum) of values above the detection limits and percentage of zeros for each group

*IL-17* interleukin-17, *CD40L* cluster of differentiation 40 ligand, *CSF* cerebrospinal fluid, *SRMA A* steroid-responsive meningitis-arteritis in acute stage, *SRMA R* recurrence of SRMA, *SRMA T* SRMA patients under treatment

### CSF and serum concentrations of CD40L

CD40L concentrations in CSF were overall very low; a high number of values were found under the detection limit. These values are described as “zero” values and are expressed as percentage for each group.

The lowest values were found in the SRMA T group, followed by the healthy group. Measurable CD40L concentrations and lower percentage of zeros were found in the SRMA A and SRMA R groups. Statistically significant differences occurred between the SRMA A (median 0.51 ng/mL; percentage of zeros 50%) and the SRMA R (median 0.22 ng/mL; percentage of zeros 33.33%) groups when compared with the SRMA T (*p* < 0.0001 and *p* = 0.0005, respectively) and healthy dogs (*p* = 0.0042 and *p* = 0.0145, respectively). CD40L concentrations in serum of all the groups examined did not differ significantly, all values were above the detection limit.

CD40L concentrations in CSF and serum as well as the percentage of zeros are summarized in Table 2. Significant differences among groups are displayed in Fig. 2.

### Correlation analysis

CSF concentrations of IL-17 and CD40L showed a strong positive correlation among each other (rSpear = 0.6601; *p* < 0.0001) and with the degree of pleocytosis in all groups (rSpear = 0.8842; *p* < 0.0001 and rSpear = 0.6649; *p* < 0.0001, respectively). In SRMA A and SRMA R, correlations between IL-17 levels in CSF and degree of pleocytosis were rSpear = 0.7574 (*p* < 0.0001) and rSpear = 0.8857 (*p* = 0.0188), respectively.

### Blood samples for PBMCs used for determination of IL-17 and IFN-γ by ELISpot

After complete diagnostic workup was made, the patients were divided in seven groups. The number of PBMCs samples used for determination of IL-17 and IFN-γ are shown in Table 3.

**Table 3:** Number of IL-17 and IFN-γ spot-forming cells (median and range)

Groups		IL-17	IFN-γ
SRMA A (*n* = 18)	Median	12.83	43.83
	Range	(3.33–34.67)	(1.67–315.00)
SRMA T (*n* = 9)	Median	13.00	25.33
	Range	(1.00–95.33)	(5.33–169.67)
Healthy (*n* = 16)	Median	4.34	291.50
	Range	(0.67–14.00)	(77.67–695.00)
Idiopathic epilepsy (*n* = 10)	Median	28.33	109.17
	Range	(2.33–66.00)	(35.33–350.00)
MUO (*n* = 8)	Median	13.50	87.50
	Range	(3.33–74.67)	(20.33–296.67)
IVDH (*n* = 7)	Median	7.67	61.00
	Range	(5.67–44.67)	(12.00–215.33)
Miscellaneous (*n* = 8)	Median	6.84	42.50
	Range	(1.67–33.00)	(14.00–369.00)

*SFCs* spot-forming cells, *IL-17* interleukin-17, *IFN-γ* Interferon gamma, *SRMA A* steroid-responsive meningitis-arteritis in acute stage, *SRMA T* SRMA patients under treatment, *MUO* meningoencephalitis of unknown origin, *IVDH* intervertebral disc herniation

### Number of PBMCs secreting IL-17 and IFN-γ

The lowest values were found in the healthy group (median 4.34 SFCs; range 0.67–14.00 SFCs), which differed significantly from SRMA A (*p* = 0.0007), SRMA T (*p* = 0.0134), idiopathic epilepsy (*p* = 0.0006), MUO (*p* = 0.0106), and intervertebral disc herniation (IVDH) (*p* = 0.0266). The number of IL-17-producing PBMCs in the epilepsy group and in cases with MUO varied widely.

IFN-γ was mostly produced by PBMCs of the healthy group (median 291.50 SFCs; range 77.67–695.00 SFCs). Significant differences were found between this group and all the other groups: SRMA A (*p* < 0.0001), SRMA T (*p* = 0.0005), idiopathic epilepsy (*p* = 0.0057), MUO (*p* = 0.0077), IVDH (*p* = 0.0029), and miscellaneous (*p* = 0.0053).

**Fig. 3:**
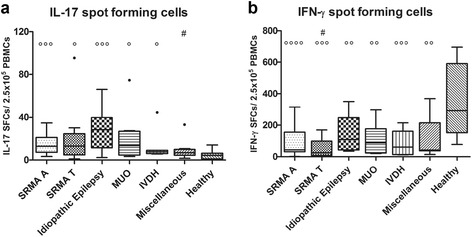
IL-17 (**a**) and IFN-γ (**b**) spot-forming cells. *Boxes* contain values from the first to the third quartile, *lines inside the box* indicate median values, and *endpoints of vertical lines* represent minimum and maximum values, and • represent outliners. *Circles* indicate statistically significant differences from the healthy group (^oooo^ *p* < 0.0001, ^ooo^ *p* < 0.005, ^oo^ *p* < 0.01, ^o^ *p* < 0.05). *Number sign* indicates statistically significant differences between idiopathic epilepsy (#*p* < 0.05) and other groups. SFCs: spot-forming cells, IL-17: interleukin-17, IFN-γ: interferon gamma, SRMA A: steroid-responsive meningitis-arteritis in acute stage, SRMA T: SRMA patients under treatment, MUO: meningoencephalitis of unknown origin, IVDH: intervertebral disc herniation

The number of IL-17 and IFN-γ SFCs are summarized in Table 3. Significant differences between groups are displayed in Fig. 3.

## Discussion

In the current study, we could support the hypothesis that SRMA is associated with a Th17-skewed immune response. Increased intrathecal levels of IL-17 and CD40L in patients with SRMA in the acute stage and during relapses were detected. Furthermore, IL-17 CSF levels showed a strong positive correlation with the degree of pleocytosis (rSpear = 0.8842; *p* < 0.0001) suggesting that IL17 might be involved in the massive migration of neutrophils in the CSF and the induction of vascular damage.

SRMA is a primary immune-mediated meningeal disorder [3], but despite extensive research, the exact etiopathogenesis remains unknown. The treatment of choice are long-term glucocorticosteroids and when dogs in the acute stage of the disease are treated promptly, the prognosis is fair to good [2, 6]. Nevertheless, a recent study showed relapses in about 30% of the cases where a lasting improvement is not achieved [39] or the required dosage of glucocorticosteroids may lead to severe side effects. Autoimmune diseases, both in canines and humans, urge the development of new treatment strategies with a specific mechanism of action to avoid potentially serious side effects. In depth understanding and characterization of the cytokine profile leading to a dysregulation of the immune system in autoimmune inflammatory disorders allows implementation of novel therapeutic approaches based on specific cytokine modulation. To date, no studies have been performed on the recently discovered IL-17-producing T cell subset (Th17) in SRMA patients. However, IL-17-producing cells have an important role in the development of several autoimmune diseases in humans such as systemic lupus erythematosus [40], rheumatoid arthritis [41], bronchial allergy [21], inflammatory bowel disease [20], multiple sclerosis [42], and Kawasaki disease [43] and also in experimental models such as collagen-induced arthritis [19] and experimental autoimmune encephalomyelitis (EAE) [22]. The extrapolation of findings in experimental rodent models for human inflammatory diseases is very limited. SRMA as a naturally occurring animal model for human meningitis and vasculitides of unknown origin including Kawasaki Syndrome [14, 15, 44, 45] might overcome this limitation. Recently, IL-17-producing cells were found in inflamed tissues of several chronic idiopathic disorders in dogs including inflammatory bowel disease, gingivitis, chronic idiopathic rhinitis, and chronic dermatoses [46]. Increased levels of IL-17 were found in dog brain tissues with granulomatous meningoencephalomyelitis (GME) [47]. Additionally, in the current study, we could show increased values of IL 17 and CD40L in serum and CSF of dogs with SRMA, most probably having a role in the massive invasion of neutrophils to the subarachnoidal space and being involved in the development of the striking vasculitis.

IL-17 is upregulated both systemically and intrathecally in patients suffering from SMRA in the acute phase and during relapses. IL-17 was measurable in all CSF samples belonging to these two groups in contrast to the control groups. Furthermore, most measurements in CSF samples of SRMA A (median 901.77 pg/mL) and SRMA R (median 533.01 pg/mL) groups exceeded the maximal detectable value for IL-17 (500 pg/mL) of the ELISA kit and had to be diluted to obtain readable values. Patients in the SRMA A and SRMA R groups displayed a neutrophilic pleocytosis. In line with the known ability of IL-17 to induce neutrophil recruitment into sites of inflammation, the groups that displayed the highest CSF IL-17 levels showed also the highest numbers of neutrophils. Furthermore, the CSF concentrations of IL-17 showed a strong positive correlation with the degree of pleocytosis (rSpear = 0.8842; *p* < 0.0001).

Strikingly, IL-17 values measured in CSF exceeded 20 times the amount of IL-17 detected in the serum of patients in the SRMA A group and more than 10 times of patients in the SRMA R group supporting the hypothesis that IL17 is produced intrathecally and not a spill over through a damaged blood-brain barrier. Although vascular lesions may be present in the coronary arteries and other organs [45, 48, 49], the vasculitis in SRMA patients is accentuated in the subarachnoidal space of the cervical meninges [7, 15]. All CSF samples in our patients were collected from the cisterna magna near to the main lesions in SRMA. Circulating cytokines reach high concentrations at the site of release, but after dilution in the blood, much lower concentrations can be found peripherally [50] as observed in the current study.

SRMA-affected dogs have higher number of helper CD4+ T cells than cytotoxic CD8+ T cells in the peripheral blood [15]. This predominance of T helper (Th) lymphocytes indicates that in SRMA patients a humoral immune response is present, occurring typically to eliminate extracellular pathogens or in autoimmune diseases [15]. Naive CD4+ T cells differentiate into different subsets with distinct effector functions when activated in a specific cytokine environment [51]. Initially, Th differentiation was proposed to be divided in two subpopulations based on their cytokine expression profiles: Th1 and Th2 [52]. Th1 synthesize IFN-γ and mediate protection against intracellular pathogens [51]. In former studies, this cytokine was only detected in low levels in SRMA [17], we confirmed this finding in the current study, demonstrating low IFN-γ spot-forming cells in dogs with SRMA. Th2 cells synthesize IL-4, IL-13, and IL-25, moderate the clearance of extracellular pathogens [51], and were found to occur in SRMA [17]. However, the paradigm of two Th subpopulations was challenged following the discovery of a third subset of Th cells, known as Th17 cells [53], which synthesize IL-17 and are potent inducers of autoimmunity and tissue inflammation [51]. Th17 cells require both TGF-ß_1_ and IL-6 for their development [54, 55]. In humans, IL-23 is essential for the maturation of inflammatory Th17 cells [22]. It has been shown that IL-6-deficient mice fail to develop a Th17 response and are resistant to the development of EAE [55, 56] and collagen-induced arthritis [57]. In recent studies, increased levels of IL-6 and TGF-ß_1_ were described in CSF of canines suffering from SRMA. A combined intrathecal increase of these proteins could induce CD4+ progenitors to differentiate into Th17 subset and enhance the autoimmune response [14]. Additionally, dendritic cells activated by Toll-like receptor (TLR) 4 and TLR9, produce IL-23, which subsequently can activate CD4^+^ T cells shifting towards Th17 differentiation under the effect of IL-6 and TGF-ß_1_ [5]. In SRMA, such an influence of Toll-like receptors was already shown [5] and furthermore supports the hypothesis of a Th17-skewed immune response in the described disease.

IL-17 has been designated IL-17A to indicate that it is the founding member of the IL-17 cytokine family consisting of IL-17A-F members [27, 58] playing an active role in inflammatory responses and in autoimmune diseases [29]. IL-17 is a pro-inflammatory cytokine secreted primarily by activated T cells [23, 24, 29, 30, 58, 59], although it is also produced by neutrophils [24], eosinophils [25], and monocytes [26]. IL-17 acts on the IL-17 receptor (IL-17R) expressed on the cells of all the tissues examined to date [27]. The activation of IL-17R results generally in the induction of other pro-inflammatory cytokines [27]. IL-17 induces the release of IL-6 and other cytokines that trigger an inflammatory reaction characterized by neutrophil influx [21] and promotes granulopoiesis [28]. Experimental studies showed that overexpression of IL-17 in the joint space of mice with collagen-induced arthritis leads to increased neutrophil recruitment [60]. Interestingly, the mechanisms by which IL-17 disrupts the tight junctions between the endothelial cells of the blood-brain barrier (BBB) were recently demonstrated and the ability of Th17 cells to transmigrate efficiently across the BBB was proven [30]. Expression of IL-17 and IL-22 receptors on the endothelial cells of the BBB results in the binding of Th17 cells to the BBB tight junctions which causes its disruption and allows Th17 cells to transmigrate across the BBB [61]. Furthermore, IL-17 enhances inflammatory cytokine production by microglia and even its synthesis by microglia and astrocytes has been proven [62]. Altogether, these mechanisms might explain the striking increase of IL-17 intrathecally and its influence on the massive invasion of neutrophils into the subarachnoidal space in dogs affected with SRMA.

Patients in the SRMA T group were under long-term treatment with prednisolone as previously described [35] and did not show any clinical signs at the time of sampling and had CSF parameters in physiological ranges. IL-17 levels in these patients were both low in CSF (median 29.67 pg/mL) and in serum (median 21.04 pg/mL). Glucocorticoids are known to induce lymphocyte apoptosis and alter leukocyte migration and redistribution. The most important immunosuppressive effect of glucocorticosteroids is on T cell activation by inhibition of the genes that encode for cytokine production [63]. The mechanisms of action of glucocorticoids are, however, unspecific and are associated with potentially serious side effects [63, 64]. In SRMA, management of the clinical signs and a complete remission is often achieved with long-term treatment with prednisolone [2]. In refractory cases, other immunosuppressive drugs are used in combination with glucocorticoids [2, 39]. However, in approximately 30% of the patients a lasting improvement is not achieved, relapses are persistent [39], and unacceptable side effects appear [64]. In humans, up to 30% of patients treated for inflammatory diseases develop glucocorticoid resistance [64]. Hence, novel therapeutic strategies targeting specific aspects of the immune response are practiced in human medicine. Current medical trials aimed to neutralize IL-17 or IL-23 have shown to be highly effective in humans in the treatment of psoriasis and show promising initial results in ankylosing spondylitis and multiple sclerosis [65]. These innovating therapies are now appearing in companion animal medicine [66] and should be considered in inflammatory autoimmune diseases like SRMA.

In the second part of our study, increased levels of soluble CD40L in CSF of dogs with SRMA A (median 0.51; range 0.16–1.95 ng/mL) and SRMA R (median 0.22; range 0.17–0.91 ng/mL) were found when compared with all other groups. Interestingly, CD40L was not measurable in any CSF sample of the SRMA T group and only in one sample of the healthy (0.17 ng/mL) and one of the miscellaneous (0.67 ng/mL in meningoencephalitis of unknown origin) groups. To our knowledge, serum and CSF values of CD40L have not been previously evaluated in canines, and hence no reference values have been established. It was unexpected to find no differences in the expression of CD40L in serum among any of the groups.

The CD40 ligand or CD154 is a transmembrane glycoprotein from the tumor necrosis factor α (TNF α) family primarily expressed on the surface of activated CD4+ T cells which interacts with the CD40 receptor expressed principally in B cells but also in other cells [31]. Activated T cells not only express CD40L in their membranes but also produce a soluble form of CD40L, which is biologically active and interacts also with the CD40 receptor [32]. The CD40 receptor is expressed by the vascular endothelial cells and its activation by CD40L leads to leukocyte adhesion [33].

Our results showed increased levels of CD40L in CSF in dogs in the acute stage and during relapses of SRMA. High levels of soluble CD40L can regulate CNS inflammation at the BBB [67]. Recently, increased expression of CD40L in CD4+ T cells as well as soluble CD40L was found in patients with Kawasaki disease and the levels correlated with coronary artery lesions [68].

The third part of our study aimed to confirm IL-17 secretion by PBMCs at the single cellular level by ELISpot assays. The development of novel tools allows us to study cell-mediated immunity in a more sensitive and precise manner. Since its development in 1983 [69], the ELISpot assays have been employed in the identification of cytokine-producing cells at the single-cell level allowing the visualization and quantification of the secretory products of individually activated cells. The dual ELISpot allows simultaneous detection of two cytokines. We performed ELISpot assays to detect IL-17 (indicating a Th17 response) and IFN-γ (indicating a Th1 response) in PBMCs after cryopreservation. Previous studies in humans have shown full functionality after cryopreservation of PBMCs [70]. Our results proved IL-17 and IFN-γ production of activated and responsive PBMCs after cryopreservation in canines. IL-17 is produced by PBMCs and not only by neutrophils occurring in high numbers in CSF samples of dogs with SRMA.

Given the systemic nature of SRMA [17, 71], PBMCs were isolated and IL-17 production was evaluated at a single-cell level by an ELISpot assay. Although, a higher number of IL-17 SFCs were found in the SRMA A group than in the healthy group, an increased production of IL-17 in PBMCs was also found in all the other groups when compared with the healthy controls. Intriguingly, single patients of the group with idiopathic epilepsy showed more spot-forming cells as found in all other diseases. Interictal increased levels of IL-17 in CSF and serum have been found in humans with three types of epilepsy (temporal lobe epilepsy, extra-temporal lobe epilepsy, and idiopathic generalized epilepsy) [72]. Another group found a significant correlation among increased IL-17 levels and seizure frequency, IL-17 is known to facilitate Th17 migration across the BBB [73]. Another explanation could be the presence of single cases with immune-mediated diseases among our idiopathic epilepsy group. In humans, some seizure disorders once thought to be idiopathic now seem to be immune-mediated [74] and occurrence of autoantibodies to different neuronal structures were found [74–76]. Nonetheless, we have clearly demonstrated the IL-17 production at the single-cell level in the peripheral blood in SRMA patients and compared it to positive and negative controls.

Finally, IFN-γ spot-forming cells in SRMA patients in the acute as well as those under treatment remained low. Previous studies demonstrated a downregulation of IFN-γ mRNA expression by means of reverse transcriptase real-time polymerase chain reaction in dogs with SRMA in the acute phase [17]; our findings confirmed low IFN-γ expression at the protein level compared to healthy dogs excluding a Th1 response and further supporting a Th17 response.

The current study is, to our knowledge, the first one to demonstrate IL-17 expression in canine CSF and its involvement in the pathogenesis in SRMA patients. Our findings follow up the already known roles of cytokines involved in the pathogenesis of SRMA [77] and give a clear direction for future treatment approaches.

## Conclusions

The described results imply that Th17 cells are inducing the autoimmune response in SRMA and are involved in the development of the severe neutrophilic pleocytosis and disruption of the BBB. The investigation of the role of IL-17 and CD40L in SRMA adds to the knowledge of pathophysiological mechanisms in SRMA and opens the discussion about new therapeutic strategies in this valuable naturally occurring large animal model.

## Abbreviations

BBB: Blood-brain barrier
CD: Cluster of differentiation
CD40L: Cluster of differentiation 40 ligand
CNS: Central nervous system
CSF: Cerebrospinal fluid
EAE: Experimental autoimmune encephalomyelitis
EDTA: Ethylenediaminetetraacetic acid
ELISA: Enzyme-linked immunosorbent assay
ELISpot: Enzyme-linked immunospot assay
IE: Idiopathic epilepsy
IFN-γ: Interferon gamma
IgA: Immunoglobulin A
IL: Interleukin
IVDH: Intervertebral disc herniation
MRI: Magnetic resonance imaging
MUO: Meningoencephalitis of unknown origin
PBMCs: Peripheral blood mononuclear cells
PBS: Phosphate-buffered
rSpear: Spearman’s rank correlation coefficient
SFCs: Spot-forming cells
SRMA: Steroid-responsive meningitis-arteritis
TGF-ß_1_: Transforming growth factor *beta* 1
Th17: T helper 17
